# Patient Perspectives on Non-Hodgkin Lymphoma: A Qualitative Study to Guide Selection of Clinical Trial Endpoints

**DOI:** 10.3390/curroncol33070427

**Published:** 2026-07-17

**Authors:** Amy Clark, Sophie Van Tomme, Lucinda Hetherington, Carla Dias Barbosa, Paul Cordero

**Affiliations:** 1 PPD™ Evidera™ Patient-Centered Research, Thermo Fisher Scientific, London W6 8BJ, UK; amy.clark2@thermofisher.com (A.C.); lucy.hetherington@thermofisher.com (L.H.); carla.dias-barbosa@thermofisher.com (C.D.B.); 2 Sanofi, 1105 BP Amsterdam, The Netherlands; sophie.vantomme@sanofi.com (S.V.T.); 3 Sanofi, Reading RG6 1PT, UK

**Keywords:** diffuse large B-cell lymphoma, mantle cell lymphoma, non-Hodgkin lymphoma, qualitative interviews, patient experience data, patient-reported outcomes, quality of life

## Abstract

The literature on the qualitative experiences of patients with non-Hodgkin lymphoma (NHL) is limited. Qualitative interviews were conducted to investigate participants’ experiences with two types of NHL (diffuse large B-cell lymphoma [n = 20] and mantle cell lymphoma [n = 10]) and evaluate the comprehensiveness of patient-reported outcome (PRO) measures. Fatigue, tiredness, body aches, night sweats, lethargy, headache, appetite loss, altered taste, and weakness were the most frequent and bothersome symptoms. Key impacts were decreased physical performance, restricted activity, sadness, distress, fear of recurrence, and worry about future. Most participants expressed positive opinions about the European Organisation for Research and Treatment of Cancer Quality of Life Questionnaire–Core 30 (EORTC QLQ-C30) (n = 22/28), EORTC QLQ-NHL-High Grade Module 29 (EORTC QLQ-NHL-HG29) (n = 12/16), EORTC QLQ-NHL-Low Grade Module 20 (EORTC QLQ-NHL-LG20) (n = 8/12), and Functional Assessment of Cancer Therapy–Lymphoma (FACT-Lym) (n = 10/14), considering them relevant to their experiences (22/27, 13/15, 9/13, and 10/12, respectively). All measures adequately captured their experiences with NHL (QLQ-C30: n = 26/26, NHL-HG29: n = 14/14, NHL-LG20: n = 11/11, and FACT-Lym: n = 12/13). These findings provide a valuable framework for informing the selection of appropriate PRO measures in NHL clinical trials and identifying potentially meaningful trial endpoints.

## Simple Summary

Non-Hodgkin lymphoma is a type of blood cancer that affects the body’s immune system. However, there is limited information on how non-Hodgkin lymphoma is impacting the life of patients. This study interviewed 30 patients with non-Hodgkin lymphoma to understand their experience with the disease, including which symptoms bothered them the most and how cancer affected their daily lives. During the interviews, health questionnaires about patient experience were also discussed. The most reported symptoms were tiredness, body aches, night sweats, and headaches. These symptoms made daily life harder by limiting physical activity, causing sadness, fear, and worry about the future. Most participants found the health questionnaires helpful and easy to use. These findings help researchers and doctors understand the importance of patient experiences with non-Hodgkin lymphoma and support the use of appropriate health questionnaires in future studies.

## 1. Introduction

Non-Hodgkin lymphoma (NHL) is a heterogeneous group of malignancies affecting immune cells [1]. Diffuse large B-cell lymphoma (DLBCL; 32%) is the most common subtype of NHL, followed by follicular lymphoma (17%), marginal zone lymphoma (8%), and mantle cell lymphoma (MCL; 4%) [2]. In 2025, the American Cancer Society estimated 80,350 new cases of NHL and 19,390 deaths attributable to the disease in the United States [3]. Recent advancements in treatment, particularly with novel targeted therapies, have significantly improved survival outcomes for patients with NHL [4]. However, many survivors continue to experience poor health-related quality of life (HRQoL) [5,6,7]. Furthermore, like other hematologic malignancies, patients with NHL frequently experience unmet care needs, particularly those related to informational support, emotional well-being, physical support, daily functioning, and family life or relational challenges [8,9].

Patient-reported outcomes (PROs) have emerged as a key tool for incorporating patient perspectives in oncology research and practice [10], with strong endorsement from regulatory institutions [11,12]. According to the Food and Drug Administration (FDA) guidance issued in June 2020, patients are the most reliable source for describing the ongoing effects of their condition and its treatment [13]. Consequently, there is a growing expectation for clinical trials to integrate patient perspectives through PROs, complementing clinical outcomes [14].

Although the significance of PROs is well established, the qualitative literature on the experiences of patients with NHL is limited. This study aimed to identify and describe the signs/symptoms and impacts from participants’ perspectives and to develop a conceptual model for two types of NHL: DLBCL and MCL. Furthermore, it sought to evaluate the readability, relevance, and comprehensiveness of four PRO measures: the European Organisation for Research and Treatment of Cancer Quality of Life Questionnaire–Core 30 (EORTC QLQ-C30), EORTC QLQ-NHL-High Grade Module 29 (EORTC QLQ-NHL-HG29), EORTC QLQ-NHL-Low Grade Module 20 (EORTC QLQ-NHL-LG20), and Functional Assessment of Cancer Therapy–Lymphoma (FACT-Lym).

## 2. Materials and Methods

### 2.1. Study Design and Patient Population

This was a non-interventional, cross-sectional, qualitative study comprising concept elicitation and cognitive debriefing interviews. Adult patients (aged ≥ 18 years) in the United States with clinician-confirmed DLBCL or MCL diagnoses who were not receiving active treatment at the time of interviews and were willing to participate in a 60 min telephonic or web-assisted telephonic interview were enrolled between 6 June and 8 September 2023. The exclusion criteria included other malignancies; HIV or other disseminated diseases; major debilitating conditions; and any clinically relevant medical conditions, such as severe comorbidities, mental illness, substance abuse, or cognitive impairment. A purposive sampling method was used to select the participants. The study was approved by the Western Copernicus Group Institutional Review Board (WCG IRB, ID45355180), a US-based IRB.

### 2.2. Concept Elicitation

Concept elicitation interviews aimed to explore participants’ experiences with NHL and update the disease-specific conceptual model of NHL previously derived from a literature review. The review was conducted in November 2020 using the Medline, PsycINFO, and Embase databases. The search strategy combined thesaurus terms and free-text keywords related to “Diseases” and “Qualitative Research”, limiting the results to studies published in English with available abstracts. The review identified key concepts that described patients’ experiences with NHL by examining 15 qualitative studies (Figure S1 and Table S1) and developing a preliminary conceptual model.

Qualitative interviews were conducted by a trained, experienced researcher (AC) with vast experience in qualitative research. The interviewer had no relationship with the study participants. A semi-structured qualitative interview guide was used to facilitate discussions during the interviews. The interview guide comprised open-ended questions designed to obtain detailed information about symptom experiences and the impact of NHL on patients’ lives. If participants did not spontaneously mention specific concepts, such as key symptoms and impacts outlined in the preliminary conceptual model, the interviewer used probes provided in the interview guide to explore and confirm these concepts. No repeat interviews were conducted, and the participants did not provide feedback on the overall findings.

### 2.3. Conceptual Model Development

The preliminary conceptual model developed through a targeted literature review was updated based on the findings from the concept elicitation interviews conducted in this study. The updated model incorporated additional signs/symptoms for the DLBCL and MCL subgroups as well as the impacts on the overall NHL population that were not included in the preliminary conceptual model.

### 2.4. PRO Instruments

Based on the review of the literature and preliminary conceptual model, EORTC QLQ-C30, EORTC QLQ-NHL-HG29, EORTC QLQ-NHL-LG20, and FACT-Lym were identified as possible measures to support the endpoint strategy of future NHL clinical trials. The EORTC QLQ-C30 (Version 3) assesses physical, psychological, and social functioning as well as the global health status and disease-related symptoms in patients with cancer (Table S2) [15]. The EORTC QLQ-NHL-HG29 and EORTC QLQ-NHL-LG20 are disease-specific QoL questionnaires that supplement the EORTC QLQ-C30 and assess the QoL of patients with high-grade and low-grade NHL, respectively [16]. The FACT-Lym (Version 4) is a disease-specific instrument that assesses the QoL of patients with lymphoma [17].

### 2.5. Cognitive Debriefing

The cognitive debriefing section of the interview evaluated the readability, relevance, and comprehensiveness of the EORTC QLQ-C30, EORTC QLQ-NHL-HG29, EORTC QLQ-NHL-LG20, and FACT-Lym. This process included a “think aloud” exercise [18], wherein participants read aloud each instruction, item, and response option from the PRO measures. The participants were encouraged to verbalize their thought process while selecting their responses.

### 2.6. Data Analysis

The data collected from the interview participants, including sociodemographic and clinical characteristics via an online questionnaire, were summarized using descriptive statistics. Key concepts were defined as those reported by ≥50% of participants with a mean disturbance rating of ≥5 on a scale of 0 (not at all bothersome) to 10 (very bothersome). These criteria align with FDA Patient-Focused Drug Development (PFDD) guidance, which emphasizes identification of concepts that are both commonly experienced and important to patients [19], and are consistent with established methods in qualitative PRO research and concept elicitation studies [20,21,22]. A saturation table, which displays the point at which no new concepts are mentioned by participants, was developed to track the concepts of interest (signs/symptoms and impacts). All interviews were audio recorded and transcribed verbatim. The transcripts were not returned to participants for comment or correction. The data analysis followed a prespecified qualitative analysis plan that included a structured coding dictionary. The coding framework was developed based on the interview guide, and draft table templates were created to standardize the presentation of the findings. Interview transcripts were coded using a granular approach that preserved patient-reported terminology and distinctions. Related concepts were grouped into broader domains in the final conceptual model while maintaining granular coding in the detailed results to preserve the richness of patient-reported experiences. AC and LH coded the data using Microsoft Word and reviewed the transcripts and coding. To ensure coding reliability, inter-coder agreement (ICA) was monitored throughout the coding process, with a target threshold of ≥80% agreement achieved before independent coding proceeded. De-identified transcripts were analyzed using the ATLAS.ti (Scientific Software Development, Berlin, Germany) (version 22).

## 3. Results

### 3.1. Participant Characteristics

A total of 30 participants were interviewed: 20 patients with DLBCL and 10 patients with MCL (Table 1). The mean ± SD age of the study population was 57.8 ± 5.7 years, and nearly half of the population was female (n = 14/30, 46.7%). No eligible patient refused to participate, and none dropped out of the study. Most participants were non-Hispanic or non-Latino (n = 22/30, 73.3%) and white (n = 18/30, 60%). The most frequently reported non-NHL comorbidities were hypertension (n = 12/30, 40%), allergies (n = 11/30, 36.7%), and high cholesterol (n = 10/30, 33.3%).

### 3.2. Concept Elicitation Interviews

Overall, patients reported 58 signs and symptoms. The most frequently reported symptoms were fatigue (DLBCL: n = 18/20, 90%; MCL: n = 10/10, 100%), tiredness (DLBCL: n = 18/20, 90%; MCL: n = 8/10, 80%), body aches/pain (DLBCL: n = 17/20, 85%; MCL: n = 7/10, 70%), night sweats (DLBCL: n = 16/20, 80%; MCL: n = 8/10, 80%), and headache/migraine (DLBCL: n = 13/20, 65%; MCL: n = 8/10, 80%). Analysis of spontaneous versus probed reporting showed that body aches/pain (n = 24 spontaneous vs. n = 0 probed), fatigue (n = 21 spontaneous vs. n = 7 probed), and fever/chills (n = 13 spontaneous vs. n = 2 probed) were predominantly reported spontaneously, whereas lethargy (n = 2 spontaneous vs. n = 19 probed), weight loss (n = 3 spontaneous vs. n = 13 probed), and weakness (n = 7 spontaneous vs. n = 10 probed) were primarily elicited through targeted probing. Complete reporting by NHL subtype and reporting method for all signs and symptoms is provided in Table S3.

A total of 64 impacts were reported. The most reported impacts included limited outdoor (DLBCL: n = 19/20, 95%; MCL: n = 9/10, 90%) and indoor (DLBCL: n = 17/20, 85%; MCL: n = 10/10, 100%) activities, reduced physical performance (DLBCL: n = 17/20, 85%; MCL: n = 10/10, 100%), concerns about the future (DLBCL: n = 18/20, 90%; MCL: n = 7/10, 70%), and feeling sad or depressed (DLBCL: n = 16/20, 80%; MCL: n = 7/10, 70%). Analysis of spontaneous versus probed reporting revealed that decreased physical performance (n = 19 spontaneous vs. n = 8 probed) and limited outdoor activities (n = 19 spontaneous vs. n = 9 probed) were predominantly reported spontaneously, whereas feeling powerless (n = 0 spontaneous vs. n = 16 probed), fear of recurrence (n = 4 spontaneous vs. n = 17 probed), and emotional exhaustion (n = 5 spontaneous vs. n = 15 probed) were primarily elicited through targeted probing. Complete reporting by NHL subtype and reporting method for all impacts are provided in Table S4. Key patient quotes for the most reported signs/symptoms and impacts are provided in Table 2. Exhaustion and weakness were reported as the most disturbing symptoms, with an average disturbance rating of 8 out of 10. However, exhaustion was predominantly reported by patients with MCL and weakness by patients with DLBCL (Table S5). Fatigue and general tiredness were the second most frequently reported disturbing symptoms, with a mean disturbance rating of 7.5 out of 10 across the total sample. Patients with MCL exclusively reported three impacts with high disturbance ratings (each rated 8 out of 10): difficulties with self-care, need for support from family and friends, and changes in relationships. In contrast, patients with DLBCL identified a separate set of impacts with high disturbance ratings, including fear of recurrence, uncertainty about cancer outcomes, hair loss, and emotional exhaustion (each rated 8 out of 10) (Table S5).

**Figure 1:**
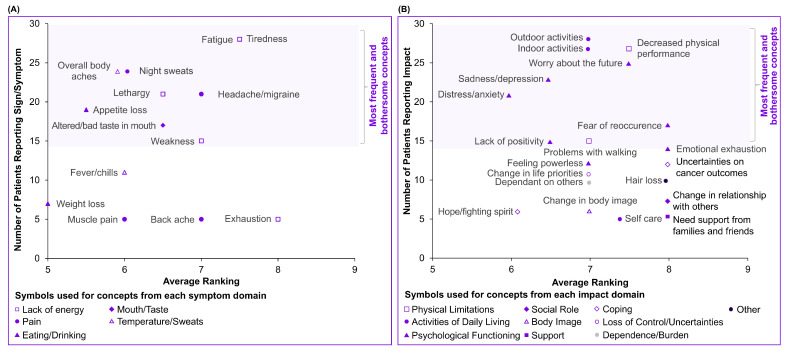
(**A**) Most frequent and bothersome symptoms of DLBCL and MCL. (**B**) Key impacts of DLBCL and MCL. DLBCL, diffuse large B-cell lymphoma; MCL, mantle cell lymphoma.

The most frequent and bothersome symptoms reported by both patients with DLBCL and those with MCL were fatigue, tiredness, body aches, night sweats, lethargy, headache/migraine, appetite loss, altered/bad taste in the mouth, and weakness (Figure 1A). Patients with DLBCL and those with MCL reported key impacts as decreased physical performance and limited indoor (housework, cleaning, or cooking) and outdoor (shopping, gardening, or going out) activities. Other key impacts were related to emotional and psychological domains, including sadness/depression, distress/anxiety, fear of lymphoma recurrence, and worry about the future (Figure 1B).

Coders achieved an ICA of 81.7%, which exceeded the pre-specified threshold of ≥80%. This confirmed adequate agreement between coders for proceeding with independent analysis of the remaining transcripts. A saturation analysis was conducted exclusively using the DLBCL group, as the sample size of the MCL group was smaller (n = 10). The conceptual saturation was achieved by the DLBCL group. In the case of the MCL group, concept occurrence was tracked chronologically, revealing that only five new concepts (6%) emerged during the final two interviews.

### 3.3. Conceptual Model

**Figure 2:**
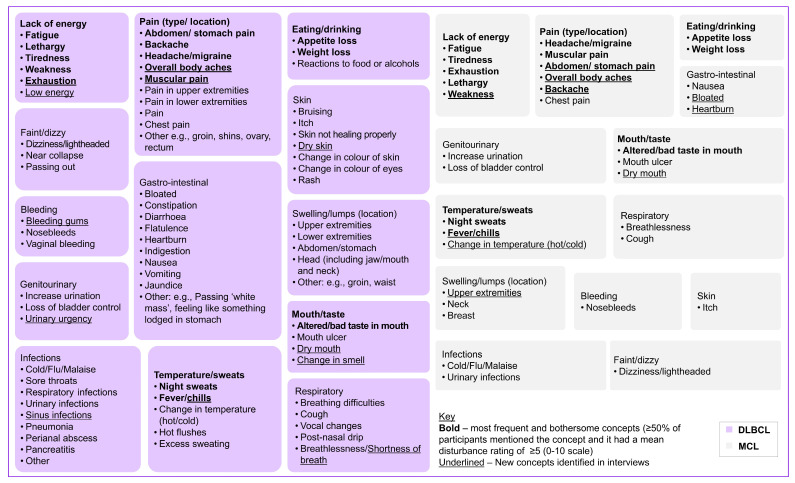
Conceptual model for signs/symptoms of DLBCL and MCL. DLBCL, diffuse large B-cell lymphoma; MCL, mantle cell lymphoma.

**Figure 3:**
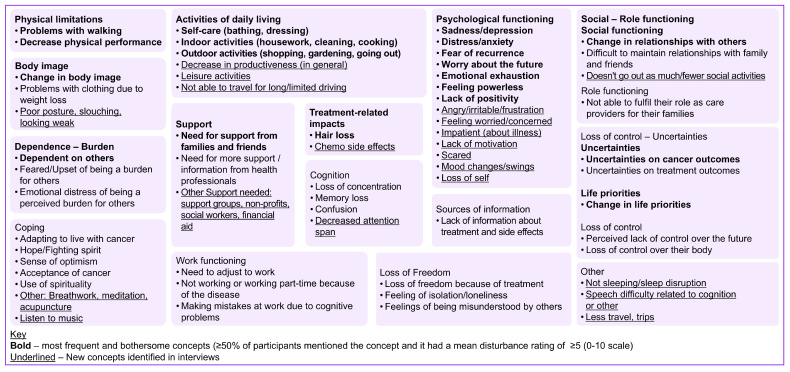
Conceptual model for impacts of NHL (DLBCL and MCL combined). DLBCL, diffuse large B-cell lymphoma; MCL, mantle cell lymphoma; NHL, Non-Hodgkin lymphoma.

A previously conducted targeted qualitative literature review revealed key symptoms and impacts experienced by patients with NHL. The concepts identified from the concept elicitation interviews were integrated with the concepts identified in the literature review to develop a comprehensive conceptual model of NHL. Therefore, 12 additional signs and symptoms were incorporated into the DLBCL population, whereas 11 were added to the MCL population (Figure 2). Regarding the impacts on QoL, 26 new impacts were identified and incorporated into the overall NHL conceptual model (Figure 3).

### 3.4. Cognitive Debriefing

A total of 28 participants (18 with DLBCL and 10 with MCL) completed the top-level debriefing for the EORTC QLQ-C30. Sixteen participants (12 with DLBCL and four with MCL) completed the EORTC QLQ-NHL-HG29 debriefing, whereas 13 participants (10 with DLBCL and three with MCL) completed the EORTC QLQ-NHL-LG20 debriefing. Fourteen participants, including 10 with DLBCL and four with MCL, completed the FACT-Lym debriefing. The mean ± SD summary score for the EORTC QLQ-C30 was 62 ± 15.5, wherein higher scores indicated better status in the functioning and global health domains but worse status in the symptom domain. The EORTC QLQ-NHL-HG29 score was 2.3 ± 1.2, with higher scores reflecting worse or increased symptoms and problems. Similarly, the EORTC QLQ-NHL-LG20 score was 41.1 ± 16.4, wherein higher scores indicated worse or increased symptoms and problems. The FACT-Lym score was 62.6 ± 13.7, with higher scores indicating a greater QoL. Collectively, these scores suggested a moderate overall health status.

Most participants (n = 22/28, 78.6%) expressed a positive opinion about the EORTC QLQ-C30, and a similar proportion (n = 22/27, 81.5%) found the measure relevant to their experiences. Additionally, all participants who responded (n = 26/26, 100%) confirmed that the EORTC QLQ-C30 adequately captured their experiences with NHL (Table 3). Most of the participants (n = 12/16, 75%) expressed a positive opinion about the EORTC QLQ-NHL-HG29. The majority (n = 13/15, 86.7%) found the items relevant to their NHL experiences, and all participants who responded (n = 14/14, 100%) indicated that the measure included all necessary concepts. More than half of the participants (n = 8/12, 66.7%) expressed a positive opinion about the EORTC QLQ-NHL-LG20. Although most (n = 9/13, 69.2%) of the participants found all items relevant, a few (n = 4/13, 30.8%) noted that several items did not apply to them. Nevertheless, all participants (n = 11/11, 100%) confirmed that the measures provided comprehensive coverage of their NHL experiences (Table 3). Most participants (n = 10/14, 71.4%) expressed a positive opinion about the FACT-Lym. Among those surveyed, a majority (n = 10/12, 83.3%) found the items relevant, and nearly all (n = 12/13, 92.3%) reported that the measure did not omit any important concepts (Table 3).

In-depth cognitive debriefing interviews were conducted for the EORTC QLQ-NHL-HG29 (DLBCL: n = 5/20, 25%; MCL: n = 3/10, 30%), EORTC QLQ-NHL-LG20 (DLBCL: n = 6/20, 30%; MCL: n = 4/10, 40%), and FACT-Lym (DLBCL: n = 5/20, 25%; MCL: n = 2/10, 20%). Overall, participants found the instructions, recall period, and response options of these instruments to be clear and easy to understand (Table S6).

## 4. Discussion

Patient perspectives on most frequent and bothersome symptoms and key impacts of NHL have not been comprehensively evaluated in previous studies. This study identified the most frequent and bothersome key symptoms and key impacts, providing new insights into patient experiences of NHL. To date, only general cancer measures, such as the EORTC QLQ-C30, have been evaluated in various trials, whereas NHL-specific measures have not yet been assessed. In this study, we evaluated the EORTC QLQ-NHL-HG29, EORTC QLQ-NHL-LG20, FACT-Lym, and EORTC QLQ-C30. The findings suggest that these instruments are suitable for defining meaningful trial endpoints in future NHL trials.

Patients with NHL frequently report fatigue as one of the most distressing symptoms [23,24,25,26]. In a qualitative study of patients with NHL undergoing chemotherapy, participants described how fatigue impaired their ability to perform routine activities. They expressed a constant struggle between their desire to perform certain activities and their unresponsive body and mind [27]. Similarly, in the current study, fatigue was reported as the most common symptom in both DLBCL (n = 18/20) and MCL (n = 10/10), with disturbance ratings of 8 and 7 out of 10, respectively. Patients described the experience as feeling completely drained of energy, with one noting, “It’s like you have nothing, like, you just don’t have any motivation whatsoever, to get up or to move around. You’re just drained, completely drained of energy.” Importantly, fatigue was among the most frequent and bothersome symptoms, along with tiredness, body aches, night sweats, lethargy, headache/migraine, appetite loss, altered/bad taste in the mouth, and weakness. Notable differences were observed in the most frequently reported symptoms between patient subgroups. Patients with DLBCL (n = 15) reported a higher incidence of weakness than that of patients with MCL (n = 2), and weakness was rated as the most disturbing symptom (8/10). In contrast, exhaustion was more frequently reported by patients with MCL (n = 5) than by those with DLBCL (n = 2) and was similarly rated as the most disturbing symptom (8/10). Reporting pattern analysis in the current study demonstrated that body aches/pain, fatigue, and fever chills were most frequently mentioned spontaneously, suggesting high clinical significance, whereas lethargy, weight loss, and weakness required probing for elicitation. Similarly, in impacts, decreased physical performance and limited outdoor activities were predominantly mentioned spontaneously, reflecting their primary importance in patients’ daily experiences. Emotional impacts required probing for elicitation, suggesting potential barriers to spontaneous disclosure of these experiences.

In a qualitative study, Cheng et al. identified several domain impairments reported by patients with DLBCL. These domains included social functioning, emotional functioning, physical functioning, cognitive functioning, role functioning, fatigue, sleep disturbance, and pain/discomfort [28]. The current study observed a few similar domain impairments in both DLBCL and MCL groups. The key domain impacts included physical functioning, activities of daily living, and psychological well-being. Although, Cheng et al. identified physical functioning as the most impacted domain in DLBCL [28], the current study found that all patients with MCL (n = 10) reported decreased physical performance as a key impact, whereas 17 of 20 patients in the DLBCL subgroup reported the same impact. These findings suggest that limitations in physical functioning are a prominent concern among patients with NHL, regardless of their subtype.

Based on the review of the literature and preliminary conceptual model, EORTC QLQ-C30, EORTC QLQ-NHL-HG29, EORTC QLQ-NHL-LG20, and FACT-Lym were identified as possible measures to support the endpoint strategy for future NHL clinical trials. The EORTC QLQ-C30 is well established in oncology clinical trials, with growing evidence supporting its utility in enhancing clinical practice [29]. Qualitative interviews with 113 patients with cancer from Europe and the United States demonstrated that the concepts assessed by the EORTC QLQ-C30 are broadly comprehensible across different language versions and relevant across cancer types and disease stages [30]. The current study also revealed a positive opinion about the EORTC QLQ-C30 among patients with NHL, who reported that the measure was relevant to their experience and adequately captured their NHL-related symptoms and impacts. Most of the ongoing trials rely on general cancer measures, such as the EORTC QLQ-C30, highlighting the need to evaluate and potentially develop NHL-specific tools to better capture PROs and demonstrate therapeutic benefit [31]. In 2017, van de Poll-Franse et al. developed NHL-specific measures for high-grade and low-grade lymphoma: the EORTC QLQ-NHL-HG29 and EORTC QLQ-NHL-LG20, respectively [16]. The current study demonstrated the relevance of these measures, with all participants reporting a positive opinion. Moreover, participants found the instructions, recall period, and response options to be clear and easy to understand. The FACT-Lym has demonstrated validity among patients with NHL and provides a targeted endpoint for NHL clinical trials [32]. Consistent with other PRO measures, the FACT-Lym was considered relevant with comprehensible instructions, recall period, and response options, with most items deemed relevant by all participants.

This study had several limitations. First, its generalizability is limited, as participants were recruited exclusively from the United States. Additionally, the study sample had a median age of 58.5 years, lower than the typical median age of 68 years at NHL diagnosis [33], which may have influenced some attributes of the conceptual model. Younger patients are more likely to be occupationally active and may differentially report impacts on work productivity, physical functioning, and social relationships compared with older, retired patients. Additionally, concerns regarding disease recurrence and long-term QoL, and symptom prominence (both frequency and intensity) may vary by age [6]. Therefore, caution should be exercised when generalizing these findings to older patient populations, and future studies should validate the conceptual model in other age groups to ensure comprehensive representation of the NHL patient experience across all age ranges. The small MCL sample size (n = 10) limited saturation analysis for this population; however, the data collected was comprehensive, offering valuable insights within the scope of our research objectives. Results were contingent on the assumption that it is reasonable to consider concepts mentioned by most patients (≥50%) with a mean disturbance rating of ≥5 represent core conceptual elements. Moreover, given the limited sample size and absence of certain subgroups, the interpretation of key concepts may be constrained. Additionally, the analysis did not account for potential selection bias due to sample characteristics (disease severity, demographics, comorbidities, coping strategies) or the impact of coding granularity on determination of key concepts, whereby broader versus more specific categorization of related concepts could influence the domains that meet the established threshold. Nearly half of the study population was female, despite NHL being more common in the male population [34]. This may further limit the generalizability of the findings, as certain symptoms, impacts, or experiences that are more prevalent or perceived differently among male patients might not have been adequately captured. Furthermore, the study sample included fewer Asian participants than anticipated, which might limit the comprehensiveness of results across diverse patient populations. While our findings highlight the importance of emotional well-being in NHL patients, the interviews and the analysis did not evaluate how clinical parameters could impact emotional status. Future research should investigate the specific relationships between clinical parameters and psychological outcomes. Treatment history and disease status data were not systematically collected in this study. While participants were relapsed or refractory to established therapies and not receiving active treatment at the time of interviews, detailed information regarding specific prior therapies, time elapsed since treatment completion, remission status, and current disease activity was not gathered. Consequently, some reported symptoms may reflect long-term treatment effects rather than disease-related manifestations, limiting definitive symptom attribution to NHL versus prior treatment exposure. Future studies should systematically collect treatment history and disease status data to enable more precise symptom attribution analysis. Finally, the interview guide was challenging to navigate within the allotted 60 min period. The concept elicitation interviews included an open-ended section for spontaneous reporting and a probing section to confirm concepts identified through a previously conducted literature review. Due to time constraints, a few responses during the cognitive interviews were incomplete, limiting the depth of data collection. Despite these limitations, the study provides valuable insights into the core concepts of interest within this patient population, which were consistent with the symptoms and impacts reported in previous research [26,27,28,35]. Furthermore, the conceptual model provides a valuable foundation to inform the selection and/or development of fit-for-purpose PRO instruments and endpoints for inclusion in NHL clinical trials.

## 5. Conclusions

This study provided extensive insights into the patient perspectives of living with NHL, specifically DLBCL and MCL. The resulting conceptual model, developed from the literature and patient interviews, effectively characterized the experiences of patients with NHL and the disease burden. These findings provide a valuable framework for informing the selection of appropriate PRO measures in NHL clinical trials and identifying potentially meaningful trial endpoints that reflect patients’ experiences of signs, symptoms, and disease impacts. Participants reported positive opinions about all evaluated PRO instruments, including the EORTC QLQ-C30, EORTC QLQ-NHL-HG29, EORTC QLQ-NHL-LG20, and FACT-Lym, confirming their perceived relevance to the NHL experience. While these findings support the potential utility of these instruments in NHL clinical trials, additional validation will be necessary to confirm their fit for purpose application and appropriate implementation in clinical trials.

## Figures and Tables

**Table 1:** Characteristics of study participants.

Characteristics	DLBCL (n = 20)	MCL (n = 10)	Total (N = 30)
Age (years)			
Mean (SD)	58.6 (5.6)	56.3 (5.7)	57.8 (5.7)
Median (Q1–Q3)	60.0 (54.0–62.5)	58.0 (51.0–60.0)	58.5 (53.0–62.0)
Sex, n			
Male	8	6	14
Female	11	3	14
Other ^1^	1	1	2
Ethnic background, n			
Hispanic or Latino	2	2	4
Non-Hispanic or non-Latino	16	6	22
Other ^2^	2	2	4
Racial background, n *			
White	14	4	18
Black or African American	1	3	4
American Indian or Alaska Native	0	0	0
Asian	0	0	0
Pacific Islander	3	0	3
Other ^3^	3 ^+^	3	6
Highest level of education, n			
Some high school	0	0	0
High school or equivalent (e.g., GED)	2	1	3
College but no degree	6	2	8
Two-year college (e.g., community or technical)	4	1	5
Four-year college	7	5	12
Graduate school	0	0	0
Other ^4^	1	1	2
Employment status, n			
Out of work for <1 year	0	1	1
On permanent disability	3	3	6
Homemaker	6	2	8
Student	0	0	0
Retired	9	3	12
Unable to work	1	1	2
Other ^5^	1	0	1
Time since diagnosis, n			
≤1 year	3	0	3
1–2 years	10	5	15
2–3 years	6	4	10
>3 years	1	1	2
Non-NHL comorbidities *, n			
Allergies	8	3	11
Arthritis	4	1	5
Depression	3	0	3
Diabetes	1	0	1
High cholesterol	7	3	10
Hypertension	9	3	12
Hypothyroidism	1	1	2
Osteoarthritis	1	0	1
None of these	3	2	5

* Responses were not mutually exclusive. Participants were allowed to select more than one response. ^+^ One participant selected both white and Pacific Islander. ^1^ Participants response: Gender does not matter these days; not needed. ^2^ Participants response: Does not matter these days; N/A; no answer; not needed. ^3^ Participants response: Does not matter these days; N/A; no answer; not needed; prefer not to disclose. ^4^ Participants response: Does not matter; no answer. ^5^ Temporary disability. DLBCL, diffuse large B-cell lymphoma; GED, general education development; MCL, mantle cell lymphoma; N, total participants; n, number of participants; NHL, Non-Hodgkin lymphoma; SD, standard deviation.

**Table 2:** Key patient quotes for the most reported signs/symptoms and impacts.

Parameter	DLBCL (n = 20)	MCL (n = 10)	Total (N = 30) [S/P]	Supporting Quotes (NHL Type, Age, Gender)
Signs/symptoms				
Fatigue	18	10	28 (21/7)	MCL, 62 years old, M: *mostly like real fatigue a lot. (…) And I’m getting to different degrees now. Like now I’m tired but it’s not as bad as like before I was getting treatment. Like before, I’d be so tired I couldn’t even move, stuff like that.*
Tiredness	18	8	26 (16/10)	MCL, 48 years old, F: *I was quite tired.*
Body aches/pain	17	7	24 (24/0)	DLBCL, 62 years old, F: *My body ached, yes, it did. Aches and pains. My body felt when you have the flu, you just feel like your body is delicate, and it’s just where you would love to just take a nice tub and get in the bath tub to warm you up and some Epson salt. Like you just, oh, it hurts.*
Night sweats	16	8	24 (17/7)	DLBCL, 51 years old, F: *Well, I get night sweats. I thought, you know, it was something else when that started.*
Headache/ Migraine	13	8	21 (15/6)	DLBCL, 66 years old, F: *What first happened is I was having a really bad headache. And the headaches were just very strong and wouldn’t relief. And it wouldn’t help when I took Tylenol or Motrin for the headache. So that’s what first started.*
Lethargy	15	6	21 (2/19)	DLBCL, 62 years old, F: *I was very lethargic.*
Weakness	15	2	17 (7/10)	DLBCL, 58 years old, M: *Just, yeah, sleepy, tired, just weak, was very weak.*
Altered/Bad taste in mouth	12	5	17 (10/7)	DLBCL, 60 years old, NR: *Sometimes something might taste sort of metallic.*
Weight loss	9	7	16 (3/13)	MCL, 58 years old, M: *I lost about 20 pounds.*
Fever/Chills	11	4	15 (13/2)	DLBCL, 49 years old, F: *Well, what my fever is, sometimes I get the chills.*
Impacts				
Decreased physical performance	17	10	27 (19/8)	DLBCL, 52 years old, F: *So I’m not as active as I used to be and I know it’s very important to me to, you know, take good care of my body and to exercise. And I was a pretty physical woman but it’s like I’ve had to cut it in half.*
Outdoor activities (shopping, gardening, or going out)	19	9	28 (19/9)	DLBCL, 58 years old, M: *I’m an avid golfer. I like to play basketball as well, and all of that stopped. […] I think, yeah, essentially, again, I was very tired and fatigued. I had the body aches. And essentially, I thought the best course of action, at that point in time, was to quit, stop those activities, based on how I was feeling, as well as to conserve energy.*
Indoor activities (housework, cleaning, or cooking)	17	10	27 (15/12)	DLBCL, 49 years old, F: *For the physical part, I’m not doing anything. I’m just sitting down or laying in the bed or in the couch, in my living room. And my family helps me. My husband helped me find books and the home, home, dishes, cooking and these things, so I’m not doing anything.*
Worry about the future	18	7	25 (13/12)	MCL, 60 years old, NR: *I would more or less worry about my future. I would worry about would my cancer get worse, stuff like that.*
Sadness/Depression	16	7	23 (10/13)	MCL, 53 years old, NR: *It’s been dropping me a little bit into the dumps, but it’s not—that’s the reality of the cancer, not necessarily did I wake up one day and just be depressed and have no idea why. […] You of course have a full purse full of other problems. You know, I mean, you’ve got—you know you’re not feeling well.*
Distress/Anxiety	15	6	21 (7/13)	DLBCL, 62 years old, M: *I worry sometimes because of lymphoma. Sometimes I get a little bit of anxiety because of my lymphoma. That has nothing to do with the treatment.*
Fear of recurrence	17	4	21 (4/17)	DLBCL, 62 years old, F: *It’s really depressing, because they have the treatments. They have the diagnostics to help find it. They have all of that jazz, but they don’t have the magic solution to cure you. And that is disheartening and unsettling. It’s worry—you worry, you’re anxious. You have moments of acceptance. You have moments of fear. It’s all over the place.*
Emotional exhaustion	14	6	20 (5/15)	DLBCL, 60 years old, F: *There’s no answer. I wasn’t told it would come back and when and how and what. I would say it’s all unknown, you know, it’s not known, so the emotions are running rampant. [..] the worry and emotion, depression is really what it boils down to. I mean, you’re not the same happy go lucky person that life is sunshine. It’s an emotion of a diagnosis.*
Feeling powerless	12	4	16 (0/16)	DLBCL, 62 years old, F: *You feel like you’ve just lost life in front of you. You’ve lost your powers.*
Lack of positivity	10	5	15 (2/13)	DLBCL, 60 years old, F: *You’re just a different person. Your mood is hindered. You don’t have the same personality. I’m not a happy go lucky, you know, friendly person. I’m a little upset, emotional. It’s—I mean, like it’s- like sure, I mean any time anybody gets an illness, and you’re told cancer, like the world is no longer you’re worried about something simple. I mean, everything kind of goes out the window. I don’t even know how to describe it.*
Problems with walking	10	5	15 (6/9)	DLBCL, 51 years old, F: *I could walk short distances but not long. […] Around the block is okay, you know. I used to walk 20 min. That was a walk to me. I can’t sustain that.*
Change in relationship with others	9	6	15 (6/9)	DLBCL, 60 years old, F: *So every single thing is affected, from outings to travel, to friends, to eating, to going out, to interactions, to friends, to your unions, to I mean everything that you have on your calendar becomes stopped. I mean, the priority gets completely changed, and it’s bolded, and that’s all that matters.*
Sense of optimism	11	7	18 (4/14)	MCL, 66 years old, F: *I mentally put positive thoughts in my head so I can get through my days, some days, where the fatigue or the tiredness is just taking over.*
Hope/Fighting spirit	11	6	17 (4/13)	MCL, 65 years old, F: *Well, when I got diagnosed and been on remission, I thought I am done, I’m good, but, when it came back again, I kind of lost my hope, you know what I’m saying. But I’m hopeful that I’m going to fight it this time too.*
Change in life priorities	11	4	15 (4/11)	DLBCL, 60 years old, F: *Yeah, it’s just your personality, your life, everything is put on a stop, and you’re living a different priority. Everything has become, you know, avoid—you’re avoiding everything, because there is a bigger problem that you’re trying to deal with that you cannot avoid. So, you know, it’s everything in your life is on hold.*

DLBCL, diffuse large B-cell lymphoma; F, female; M, male; MCL, mantle cell lymphoma; NHL, Non-Hodgkin lymphoma; NR, not reported; P, probed; S, spontaneous.

**Table 3:** PRO instruments top-level overview.

PRO Instruments		DLBCL n = 18	MCL n = 10	Total N = 28	Illustrative Quotes (NHL Subtype, Age, Gender)
EORTC QLQ-C30					
Overall impressions (n = 27)	Positive	n = 15	n = 7	n = 22	DLBCL, 62 years old, F: *It was fine. I mean, it was kind of that you’ve been asking me questions about things […] it was quite simple to answer. I wasn’t confused or at a loss of words of what to do […] it’s specific during the past week.*
	Negative	n = 0	n = 1	n = 1	MCL, 49 years old, M: *It doesn’t say what the questionnaire is about on the title. Another thing is this says during the past week. Is that starting when? This week or, because one week, is it five days, a weekday? Because a whole week is, could be seven days or is it a work week, five days. And a week ago, that would’ve been last Thursday or starting Friday, maybe. I don’t really, my memory doesn’t go all the way back that far.*
	Neutral	n = 2	n = 2	n = 4	DLBCL, 66 years old, F: *It’s just trying to gauge me on a scale of what I need or what I don’t need or how I felt in a week. It’s fine. It’s just sometimes it’s a bit repetitive, but it’s okay.*
Items that were not relevant (n = 27)	Yes	n = 3	n = 0	n = 3	MCL, 59 years old, M: *The only thing I’d say that wasn’t relevant, was when it asked about their employment related questions, and obviously, if you’re unemployed. To a greater or lesser degree, it had relevance.*
	No	n = 14	n = 8	n = 22	DLBCL, 62 years old, F: *No, everything was relevant to me.*
	Mixed response	n = 1	n = 1	n = 2	MCL, 58 years old, M: *Yeah, but it wasn’t—let me look. Something I didn’t, yeah, about work. But other people do work, so that doesn’t—that’s just me. I wouldn’t take them out of the survey. You could just not answer. That was the only one that was not appropriate. Oh, and sex. I don’t. Wasn’t there a sex question?*
Anything not covered (n = 26)	Yes	n = 0	n = 0	n = 0	-
	No	n = 18	n = 8	n = 26	MCL, 62 years old, F: *I thought it was pretty thorough.*
	Mixed response	n = 0	n = 0	n = 0	-
FACT-Lym					
Overall impressions (n = 14)	Positive	n = 7	n = 3	n = 10	DLBCL, 49 years old, F: *That was good too. I mean, they covered everything and they divided very wisely and give me different numbers for that and they asked in general. It was okay and understandable.*
	Negative	n = 2	n = 1	n = 3	MCL, 58 years old, M: *It’s a more in-depth questionnaire than the previous one… anything that I have to do additional that I don’t need to, especially because I lack energy is not something that I enjoy, so that’s my perspective, of course.*
	Neutral	n = 1	n = 0	n = 1	DLBCL, 58 years old, M: *You know, we just talked about this, you know. It’s about what was happening, the things that you asked me at the beginning.*
Items that were not relevant (n = 12)	Yes	n = 2	n = 0	n = 2	DLBCL, 60 years old, M: *There was a bunch [of items]…that didn’t apply to me…Itching, chills, sweats, fevers, just discomfort, of pain, the stomach area. And the other one was bothered by lumps or swelling in certain parts of my body.*
	No	n = 7	n = 3	n = 10	DLBCL, 62 years old, F: *I don’t believe so. I believe everything was relevant.*
	Mixed response	n = 0	n = 0	n = 0	-
Anything not covered (n = 13)	Yes	n = 0	n = 0	n = 0	-
	No	n = 8	n = 4	n = 12	DLBCL, 56 years old, M: *No, I like them. All in all, it was pretty good. Very thorough.*
	Mixed response	n = 1	n = 0	n = 1	DLBCL, 58 years old, M: *I think it all depends on the timeframe, right? In this case, we’re looking at the good in the past week, so I mean I think if you asked me in the last two weeks or a month, maybe things would be a little bit different.*
EORTC QLQ-NHL-HG29					
Overall impressions (n = 16)	Positive	n = 8	n = 4	n = 12	MCL, 48 years old, F: *I think…this just focuses on more of the overall feelings, and…it talks about the physical symptoms and the emotional symptoms. Same thing, on a gradient scale. I felt it was straightforward. It was easy to understand.*
	Negative	n = 1	n = 0	n = 1	DLBCL, 60 years old, F: *Yeah, that one has a lot of ones because it was irrelevant…I’m not having all these symptoms of coughing, dry mouth…and especially in the last week, so it depends on where I’m answering this questionnaire and at what time in the journey…but, at this time…I’m not feeling unwell. I might have some night sweats and be upset about it, but that’s really about all.*
	Neutral	n = 3	n = 0	n = 3	DLBCL, 62 years old, F: *Okay. The same thing. I mean, it’s asking same type of stuff. It’s not any different. It’s easy to answer. It’s my only complaint is why is it repetitive.*
Items that were not relevant (n = 15)	Yes	n = 1	n = 0	n = 1	DLBCL, 56 years old, M: *Didn’t have a cough, didn’t have dry mouth, didn’t have taste problems. Those were the ones that didn’t affect me.*
	No	n = 9	n = 4	n = 13	DLBCL, 49 years old, F: *No, everything was relevant. It was okay.*
	Mixed response	n = 1	n = 0	n = 1	DLBCL, 58 years old, M: *I think it’s good questions. Have you had muscle weakness…aches, pains, bones, dry cough, dry mouth? All of this is important. What didn’t seem relevant to me was I never had any tinglings in the hand or feet. I never had numbness in my fingers or toes…this doesn’t apply to me, therefore it shouldn’t be on there.*
Anything not covered (n = 14)	Yes	n = 0	n = 0	n = 0	-
	No	n = 12	n = 2	n = 14	DLBCL, 62 years old, F: *No, I think everything was covered okay.*
	Mixed response	n = 0	n = 0	n = 0	-
EORTC QLQ-NHL-LG20					
Overall impressions (n = 12)	Positive	n = 6	n = 2	n = 8	DLBCL, 52 years old, F: *Actually they’re all similar. Yeah, no problems. Simple to read, nothing confusing.*
	Negative	n = 1	n = 1	n = 2	MCL, 58 years old, M: *Basically, just wants to get my perspective of how I feel and what I’m going through and basically just more in depth than the previous one. Anything that requires effort, a lot of thinking I don’t like.*
	Neutral	n = 2	n = 0	n = 2	DLBCL,58 years old, M: *I think a little repetitive, but again, I think it’s important to—sometimes you have to ask these questions multiple times before some people, before it sinks through.*
Items that were not relevant (n = 13)	Yes	n = 4	n = 0	n = 4	DLBCL, 60 years old, M: *Have you worried about getting another type of cancer? Have you worried about treatment causing future health problems? I just put not at all.*
	No	n = 6	n = 3	n = 9	DLBCL, 58 years old, M: *To me, pretty much everything was relevant. I can relate to everything that was there.*
	Mixed response	n = 0	n = 0	n = 0	-
Anything not covered (n = 11)	Yes	n = 0	n = 0	n = 0	-
	No	n = 9	n = 2	n = 11	DLBCL, 60 years old, M: *No. I think everything’s relevant here.*
	Mixed response	n = 0	n = 0	n = 0	-

DLBCL, diffuse large B-cell lymphoma; EORTC QLQ-C30, the European Organisation for Research and Treatment of Cancer Quality of Life Questionnaire–Core 30; EORTC QLQ-NHL-HG29, EORTC QLQ-High Grade Module 29; EORTC QLQ-NHL-LG20, EORTC QLQ-Low Grade Module 20; F, female; FACT-Lym, Functional Assessment of Cancer Therapy–Lymphoma; MCL, mantle cell lymphoma; M, male; mantle cell lymphoma; NHL, Non-Hodgkin lymphoma; PRO, patient-reported outcome.

## Data Availability

The original contributions presented in this study are included in the article/Supplementary Material. Further inquiries can be directed to the corresponding author.

## Supplementary Materials

The following supporting information can be downloaded at: https://www.mdpi.com/article/10.3390/curroncol33070427/s1, References [36,37,38,39,40,41,42,43,44,45,46,47,48,49] are cited in the Supplementary Materials. Figure S1: PRISMA flow chart of targeted literature review; Table S1: Study characteristics; Table S2: Characteristics of questionnaires; Table S3: Signs/symptoms reported by patients; Table S4: Impacts reported by patients; Table S5: Disturbance Rating in DLBCL, MCL, and overall sample; Table S6: Understanding of PRO instructions, recall period, and response options with Illustrative quotes.

## Abbreviations

The following abbreviations are used in this manuscript:

NHL	Non-Hodgkin Lymphoma
DLBCL	Diffuse Large B-cell Lymphoma
EORTC QLQ-C30	European Organization for Research and Treatment of Cancer Quality of Life Questionnaire–Core 30
EORTC QLQ-NHL-HG29	EORTC QLQ-High Grade Module 29
EORTC QLQ-NHL-LG20	EORTC QLQ-Low Grade Module 20
FACT-Lym	Functional Assessment of Cancer Therapy–Lymphoma
HIV	Human Immunodeficiency Virus
HRQoL	Health-related Quality of Life
MCL	Mantle Cell Lymphoma
N	Total Participants
n	Number of Participants
PRO	Patient Reported Outcome
QoL	Quality of Life
SD	Standard Deviation
WCG IRB	Western Copernicus Group Institutional Review Board
