# Supplementary material for: Patient Perspectives on Non-Hodgkin Lymphoma: A Qualitative Study to Guide Selection of Clinical Trial Endpoints
Source: Curr Oncol. 2026 Jul 17;33(7):427. doi: 10.3390/curroncol33070427 (PMC13408806; doi:10.3390/curroncol33070427)
Supplement: Supplementary file 1 [file curroncol-33-00427-s001.zip › curroncol-4314723-supplementary.pdf]

Figure S1. PRISMA flow chart of targeted literature review

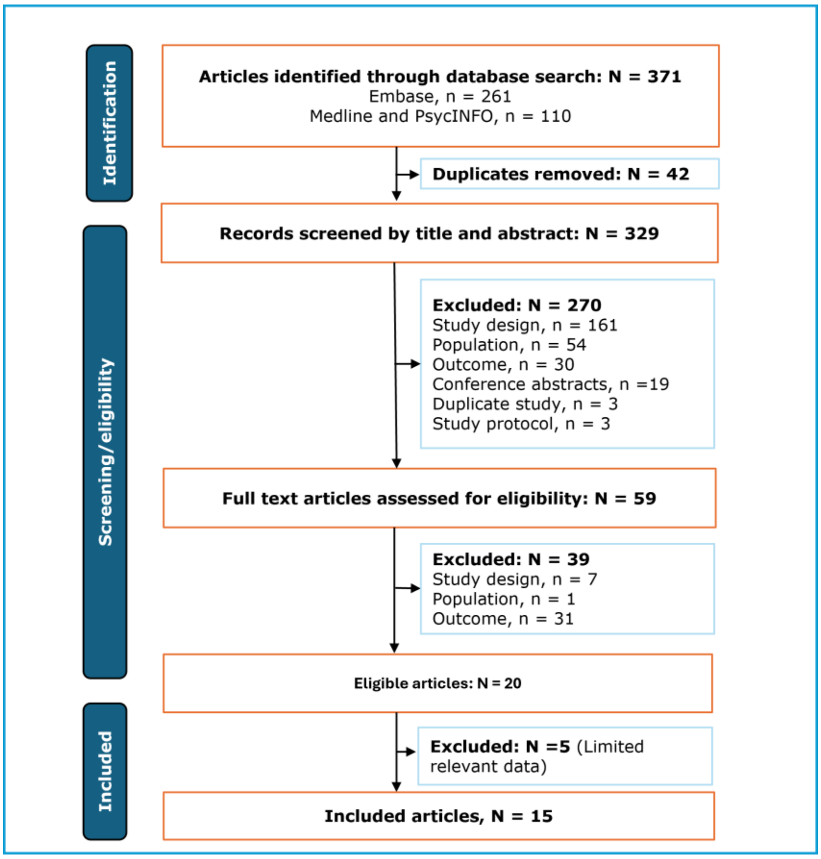

Table S1. Study characteristics

Reference	Lymphoma type (disease stage)	Topic	Sample size (n)
Yu et al. (2020) [36]	DLBCL (no information on disease stage)	Exercise barriers and facilitators during HSCT treatment	1
Hasskarl et al. (2020) [37]	DLBCL (r/r)	Expectations, underlying treatment knowledge, and concerns of patients who are awaiting chimeric antigen receptor T-cell therapy	36
Howell et al. (2019) [38]	DLBCL and HL (indolent and aggressive)	Perceptions of disease-related factors affecting time to diagnosis	DLBCL: 12<br>HL: 5
Swash et al. (2018) [39]	NHL (no information on disease stage)	Type of needs throughout patients' experiences of diagnosis, treatment, and survivorship	6
Chircop et al. (2018a) [40]	NHL (no information on disease stage)	Use of metaphors	6
Chircop et al. (2018b) [27]	Aggressive NHL	Lived experience of patients with NHL during the initial stages of chemotherapy (from commencement to mid-treatment)	6
Chircop et al. (2017) [41]	NHL (no information on disease stage)	Coping strategies and supportive care needs while undergoing chemotherapy	6

Reference	Lymphoma type (disease stage)	Topic	Sample size (n)
Broeckelmann et al. (2017) [42]	HL (advanced stage)	Drivers of treatment preferences among patients for choice of front-line treatments in advanced stage H *(no relevant concepts for physicians)*	289
Lie et al. (2017) [43]	HL and NHL survivors	Experiences with late effect-related care and preferences for long-term follow-up care	HL: 21<br>NHL: 13
Matheson et al. (2016) [44]	HL survivors	Experiences during the first year following the end of initial treatment	10
Wall et al. (2011) [45]	NHL (no information on disease stage)	Patients' experience during the period leading up to the diagnosis of NHL	28
Hess et al. (2011) [46]	HL and NHL survivors of childhood malignancy	Knowledge about diagnosis, treatment, and risk of late effects	HL: 84<br>NHL: 44
Docherty et al. (2006) [47]	B-cell HL (no information on disease stage)	Daily symptoms experienced by a teenaged girl undergoing treatment for cancer	1
Crom et al. (2005) [48]	HL survivors (favorable, i.e., stage I or II; localized and unfavorable, i.e., stage III or IV)	Perceptions of female HL survivors on current health status and future health risks, and self-care practices to prevent or diminish health risks	20
Stephens et al. (2005) [49]	NHL and other hematologic malignancies (no information on disease stage)	Lived experience of patients who have undergone autologous hematopoietic stem cell transplant	2

DLBCL, diffuse large B-cell lymphoma; HL, Hodgkin's lymphoma; HSCT, hematopoietic stem cell transplantation; NHL, non-Hodgkin lymphoma.

Table S2. Characteristics of questionnaires

Parameters	EORTC QLQ-C30	FACT-Lym	EORTC QLQ-NHL-HG29	EORTC QLQ-NHL-LG20
Number of items	30	42	29	20
Scales and assessments	• Five functional scales assessing physical, role, cognitive, emotional, and social functioning<br>• Nine multi- and single-item scales assessing fatigue, nausea and vomiting, pain, loss of appetite, diarrhea, dyspnea, constipation, insomnia, and financial impact<br>• A global health status/QoL scale	• 27 items assessing general QoL related to cancer<br>• 15 items assessing symptoms and concerns specific to lymphoma	• Four multi-item scales, including symptom burden, physical condition and fatigue, emotional impact, worries about health and functioning<br>• An item scale on neuropathy<br>• Three items are only relevant to certain individuals; these are only scored if applicable	• Four multi-item scales, including symptom burden, physical condition and fatigue, emotional impact, and worries about health.<br>• Two items are only relevant to certain individuals; these are only scored if applicable
Response options	Likert scales (4 or 7 options)	Five-point Likert-type response scale	Four-point Likert response scale	Four-point Likert response scale
Recall period	Past week	Past 7 days	Past week or past 4 weeks	Past week or past 4 weeks
Score meaning	Higher scores indicate a better status in functioning and global health domains, but a worse status in symptom domains	Higher scores indicate greater QoL	Higher scores reflect worse or increased symptoms and problems	Higher scores reflect worse or increased symptoms and problems

EORTC QLQ-C30, the European Organisation for Research and Treatment of Cancer Quality of Life Questionnaire–Core 30; EORTC QLQ-NHL-HG29, EORTC QLQ-High Grade Module 29; EORTC QLQ-NHL-LG20, EORTC QLQ-Low Grade Module 20; FACT-Lym, Functional Assessment of Cancer Therapy–Lymphoma; QoL, quality of life.

Table S3. Signs/symptoms reported by patients

Domain	Symptom	Total number of patients that reported the symptom (Spontaneously/Probed,S/P)		
		DLBCL (n=20) [total (S/P)]	MCL (n=10) [total (S/P)]	Total (N=30) [total (S/P)]
Lack of energy	Fatigue	18 (13/5)	10 (8/2)	28 (21/7)
	Tiredness	18 (11/7)	8 (5/3)	26 (16/10)
	Lethargy	15 (1/14)	6 (1/5)	21 (2/19)
	Weakness	15 (5/10)	2 (2/0)	17 (7/10)
	Exhaustion	2 (1/1)	5 (0/5)	7 (1/6)
	Low energy	5 (5/0)	0	5 (5/0)
Pain	Overall body aches/pain	17 (17/0)	7 (7/0)	24 (24/0)
	Headache/migraine	13 (10/3)	8 (5/3)	21 (15/6)
	Pain in the lower extremities (legs, feet, hips, or knees)	8 (3/5)	0	8 (3/8)
	Backache	3 (0/3)	5 (5/0)	8 (5/3)
	Abdomen/stomach pain	5 (0/5)	1 (1/0)	6 (1/5)
	Pain in upper extremities (arms, shoulders, or neck)	5 (2/3)	0	5 (2/3)
	Muscle pain	0	5 (2/3)	5 (2/3)
	Chest pain	0	2 (0/2)	2 (0/2)

Domain	Symptom	Total number of patients that reported the symptom (Spontaneously/Probed,S/P)		
		DLBCL (n=20) [total (S/P)]	MCL (n=10) [total (S/P)]	Total (N=30) [total (S/P)]
**Swelling/Lumps**	Upper extremities (neck, head, arms, or hands)	7 (7/0)	2 (2/0)	9 (9/0)
	Lower extremities (groin, legs, or ankles)	7 (5/2)	0	7 (5/2)
**Eating/Drinking**	Appetite loss/unable to eat	12 (9/3)	6 (5/1)	18 (14/4)
	Weight loss	9 (1/8)	7 (2/5)	16 (3/13)
	Reactions to food or alcohol	2 (0/2)	0	2 (0/2)
**Mouth/Taste**	Altered/bad taste in mouth	12 (7/5)	5 (3/2)	17 (10/7)
	Dry mouth	4 (4/0)	2 (2/0)	6 (6/0)
	Mouth ulcers	1 (1/0)	1 (0/1)	2 (1/1)
	Change in smell	1 (1/0)	0	1 (1/0)
**Temperature/Sweats**	Night sweats	16 (11/5)	8 (6/2)	24 (17/7)
	Fever/chills	11 (10/1)	4 (3/1)	15 (13/2)
	Change in temperature (hot/cold)	7 (3/4)	2 (1/1)	9 (4/5)
	Hot flushes	5 (0/5)	0	5 (0/5)
	Excessive sweating	5 (2/3)	0	5 (2/3)
**Faint/Dizzy**	Dizziness/lightheadedness	8 (5/3)	2 (1/1)	10 (6/4)
	Near collapse	1 (0/1)	0	1 (0/1)
**Gastrointestinal**	Nausea	9 (7/2)	2 (2/0)	11 (9/2)
	Diarrhea	9 (4/5)	0	9 (4/5)
	Bloating	4 (1/3)	1 (1/0)	5 (2/3)
	Heartburn	4 (0/4)	1 (1/0)	5 (1/4)
	Constipation	4 (1/3)	0	4 (1/3)

Domain	Symptom	Total number of patients that reported the symptom (Spontaneously/Probed,S/P)		
		DLBCL (n=20) [total (S/P)]	MCL (n=10) [total (S/P)]	Total (N=30) [total (S/P)]
	Flatulence	2 (0/2)	0	2 (0/2)
	Indigestion	2 (0/2)	0	2 (0/2)
	Vomiting	2 (1/1)	0	2 (1/1)
**Bleeding**	Bleeding gums	1 (1/0)	0	1 (1/0)
**Genitourinary**	Loss of bladder control	5 (0/5)	0	5 (0/5)
	Increased urination	2 (1/1)	1 (1/0)	3 (2/1)
	Urinary urgency	1 (1/0)	0	1 (1/0)
**Skin**	Itch	5 (2/3)	2 (0/2)	7 (2/5)
	Dry skin	4 (3/1)	0	4 (3/1)
	Bruising	3 (0/3)	0	3 (0/3)
	Skin not healing properly	1 (0/1)	0	1 (0/1)
**Respiratory**	Breathlessness/Shortness of breath	3 (3/0)	3 (2/1)	6 (5/1)
	Breathing difficulties	4 (2/2)	0	4 (2/2)
	Cough	3 (0/3)	0	3 (0/3)
	Vocal changes	2 (0/2)	0	2 (0/2)
	Post-nasal drip	1 (0/1)	0	1 (0/1)
**Neurosensory**	Visual disturbances (light sensitivity/loss/blurry vision)	2 (1/1)	1 (1/0)	3 (2/1)
	Numbness/loss of sensation	2 (2/0)	0	2 (2/0)
**Infections**	Cold/flu/malaise	6 (4/2)	1 (1/0)	7 (5/2)
	Sore throat	5 (0/5)	0	5 (0/5)
	Urinary infections	3 (2/1)	0	3 (2/1)

Domain	Symptom	Total number of patients that reported the symptom (Spontaneously/Probed,S/P)		
		DLBCL (n=20) [total (S/P)]	MCL (n=10) [total (S/P)]	Total (N=30) [total (S/P)]
	Respiratory infections	1 (0/1)	0	1 (0/1)
	Sinus infections	1 (1/0)	0	1 (1/0)

DLBCL, diffuse large B-cell lymphoma; MCL, mantle cell lymphoma; N, total population; n, number of patients; P, probed; S, spontaneous.

Table S4. Impacts reported by patients

Domain	Impact	Total number of patients that reported the impact (Spontaneously/Probed,S/P)		
		DLBCL (N=20) [total (S/P)]	MCL (N=10) [total (S/P)]	Total (N=30) [total (S/P)]
**Physical limitations**	Decreased physical performance	17 (11/6)	10 (8/2)	27 (19/8)
	Problems with walking	10 (3/7)	5 (3/2)	15 (6/9)
**Activities of daily living**	Outdoor activities (shopping, gardening, or going out)	19 (13/6)	9 (6/3)	28 (19/9)
	Indoor activities (housework, cleaning, or cooking)	17 (11/6)	10 (4/6)	27 (15/12)
	Self-care	8 (1/7)	5 (0/5)	13 (1/12)
	Not being able to travel for long/limited driving	2 (2/0)	1 (1/0)	3 (3/0)
	Decrease in productivity (in general)	2 (2/0)	0	2 (2/0)
	Leisure activities	0	1 (1/0)	1 (1/0)
**Psychological functioning**	Worry about the future	18 (7/11)	7 (6/1)	25 (13/12)
	Sadness/depression	16 (8/8)	7 (2/5)	23 (10/13)
	Distress/anxiety	15 (7/8)	6 (1/5)	21 (7/13)
	Fear of recurrence	17 (2/15)	4 (2/2)	21 (4/17)

Domain	Impact	Total number of patients that reported the impact (Spontaneously/Probed,S/P)		
		DLBCL (N=20) [total (S/P)]	MCL (N=10) [total (S/P)]	Total (N=30) [total (S/P)]
	Emotional exhaustion	14 (3/11)	6 (2/4)	20 (5/15)
	Feeling powerless	12 (0/12)	4 (0/4)	16 (0/16)
	Lack of positivity	10 (2/8)	5 (0/5)	15 (2/13)
	Angry/Irritable/Frustrated	2 (2/0)	6 (6/0)	8 (8/0)
	Feeling worried/concerned	6 (6/0)	1 (1/0)	7 (7/0)
	Mood changes/swings	4 (4/0)	0	4 (4/0)
	Impatient (about illness)	1 (1/0)	0	1 (1/0)
	Lack of motivation	1 (1/0)	0	1 (1/0)
	Scared	1 (1/0)	0	1 (1/0)
	Self-pity	0	1 (1/0)	1 (1/0)
**Body image**	Change in body image	6 (1/5)	6 (0/6)	12 (1/11)
	Problems with clothing due to weight loss	0	3 (0/3)	3 (0/3)
	Poor posture, slouching, looking weak	0	2 (0/2)	2 (0/2)
**Social/role functioning**	Change in relationship with others	9 (3/6)	6 (3/3)	15 (6/9)
	Difficult in maintaining relationships with family and friends	9 (2/7)	4 (3/1)	13 (5/8)
	Not being able to fulfill their role as caregivers/providers for their families	5 (14)	3 (0/3)	8 (14/3)
	Do not go out as much/fewer social activities	6 (6/0)	0	6 (6/0)
**Work functioning**	Not working or working part-time because of the disease	3 (2/1)	1 (0/1)	4 (2/2)
	Need to adjust to work	3 (1/2)	0	3 (1/2)
	Making mistakes at work due to cognitive problems	2 (0/2)	0	2 (0/2)
**Support**	Need support from family and friends	9 (2/7)	5 (1/4)	14 (3/11)

Domain	Impact	Total number of patients that reported the impact (Spontaneously/Probed,S/P)		
		DLBCL (N=20) [total (S/P)]	MCL (N=10) [total (S/P)]	Total (N=30) [total (S/P)]
	Need support/information from healthcare professionals	7 (1/6)	3 (2/1)	10 (3/7)
	Support needed: support groups, non-profits, social workers, and financial aid	1 (1/0)	0	1 (1/0)
Coping	Sense of optimism	11 (3/8)	7 (1/6)	18 (4/14)
	Hope/ Fighting spirit	11 (2/9)	6 (2/4)	17 (4/13)
	Adapting to living with cancer	8 (1/7)	3 (2/1)	11 (3/8)
	Acceptance of cancer	7 (2/5)	4 (0/4)	11 (2/9)
	Use of spirituality	8 (0/8)	1 (0/1)	9 (0/9)
	Breathwork, meditation, acupuncture	3 (2/1)	2 (1/1)	5 (3/2)
	Listening to music	0	1 (1/0)	1 (1/0)
Loss of control/uncertainties	Change in life priorities	11 (3/8)	4 (1/3)	15 (4/11)
	Uncertainties regarding cancer outcomes	12 (8/4)	2 (1/1)	14 (9/5)
	Perceived lack of control over the future	8 (4/4)	4 (2/2)	12 (6/6)
	Uncertainties regarding treatment outcomes	9 (3/6)	0	9 (3/6)
	Loss of control over body	6 (1/5)	2 (1/1)	8 (2/6)
	Loss of control over health	2 (1/1)	0	2 (1/1)
Dependence/burden	Dependent on others	10 (4/6)	3 (0/3)	13 (4/9)
	Fear of/ upset about being a burden on others	9 (2/7)	2 (2/0)	11 (4/7)
	Emotional distress of being perceived as a burden by others	8 (3/5)	2 (0/2)	10 (3/7)
Isolation/ Loss of freedom	Feeling isolation/loneliness	6 (2/4)	4 (0/4)	10 (2/8)
	Loss of freedom because of treatment	6 (0/6)	2 (1/1)	8 (1/7)
	Feelings of being misunderstood by others	0	1 (0/1)	1 (0/1)

Domain	Impact	Total number of patients that reported the impact (Spontaneously/Probed,S/P)		
		DLBCL (N=20) [total (S/P)]	MCL (N=10) [total (S/P)]	Total (N=30) [total (S/P)]
**Source of information**	Lack of information about treatment and side effects	2 (1/1)	0	2 (1/1)
**Cognition**	Loss of concentration	3 (0/3)	3 (2/1)	6 (2/4)
	Memory loss	1 (0/1)	1 (1/0)	2 (1/1)
	Confusion	2 (1/1)	0	2 (1/1)
	Decreased attention span	2 (2/0)	0	2 (2/0)
**Treatment related**	Hair loss	10 (4/6)	4 (3/1)	14 (7/7)
	Chemotherapy side effects	0	1 (1/0)	1 (1/0)
**Other impacts**	Not sleeping/sleep disruption	3 (3/0)	3 (3/0)	6 (6/0)
	Less travel/trips	0	4 (4/0)	4 (4/0)
	Speech difficulty	1 (1/0)	0	1 (1/0)

DLBCL, diffuse large B-cell lymphoma; MCL, mantle cell lymphoma; P, probed; S, spontaneous.

Table S5. Disturbance Rating in DLBCL, MCL, and overall sample

Symptom/impact	Mean symptom/impact disturbance rating (0–10)					
	DLBCL		MCL		Overall	
	n	Average rating	n	Average rating	N	Total average rating
Symptoms						
Exhaustion	--	--	5	8	5	**8**
Weakness	15	8	--	--	15	**8**
Fatigue	18	8	10	7	28	**7.5**
Tiredness	18	8	10	7	28	**7.5**

Symptom/impact	Mean symptom/impact disturbance rating (0–10)					
	DLBCL		MCL		Overall	
	n	Average rating	n	Average rating	N	Total average rating
Headache/migraine	13	7	8	7	21	**7**
Backache	--	--	5	7	5	**7**
Altered/Bad taste in mouth	12	6	5	7	17	**6.5**
Lethargy	15	6	6	7	21	**6.5**
Overall body aches	17	6	7	6	24	**6**
Muscle pain	--	--	5	6	5	**6**
Appetite loss	12	5	7	6	19	**5.5**
Night sweats	16	6	8	6	24	**6**
Fever/Chills	11	6	--	--	11	**6**
Weight loss	--[+]	--[+]	7	5	7	**5**
Impact						
Self-care	--[+]	--[+]	5	8	5	**8**
Need support from families and friends	--[+]	--[+]	5	8	5	**8**
Change in relationship with others	--[+]	--[+]	6	8	6	**8**
Fear of reoccurrence	17	8	--[+]	--[+]	17	**8**
Uncertainties on cancer outcomes	12	8	--[+]	--[+]	12	**8**
Hair loss	10	8	--[+]	--[+]	10	**8**
Emotional exhaustion	14	8	--[+]	--[+]	14	**8**
Decreased physical performance	17	8	10	7	27	**7.5**

Symptom/impact	Mean symptom/impact disturbance rating (0–10)					
	DLBCL		MCL		Overall	
	n	Average rating	n	Average rating	N	Total average rating
Worry about the future	18	8	7	7	25	**7.5**
Change in life priorities	11	7	--+	--+	11	**7**
Feeling powerless	12	7	--+	--+	12	**7**
Problems with walking	10	6	5	8	15	**7**
Change in body image	--+	--+	6	7	6	**7**
Dependent on others	10	7	--	--	10	**7**
Indoor activities	17	7	10	7	27	**7**
Outdoor activities	19	7	9	7	28	**7**
Hope/ Fighting spirit	--+	--+	6	7	6	**7**
Sadness/Depression	16	6	7	7	23	**6.5**
Lack of positivity	10	6	5	7	15	**6.5**
Distress/Anxiety	15	6	6	6	21	**6**

DLBCL, diffuse large B-cell lymphoma; MCL, mantle cell lymphoma.

Note: Only patients endorsing the symptoms were rated for their disturbance on a scale of 0 to 10. +Participants did not provide ranking.

Concepts reported by only one group.

Table S6. Understanding of PRO instructions, recall period, and response options with Illustrative quotes

Parameters	DLBCL N=5	MCL N=2	Total N=7	Illustrative quotes (NHL subtype, age, gender)
**FACT-Lym**				
**Overall instructions**				

Parameters	DLBCL N=5	MCL N=2	Total N=7	Illustrative quotes (NHL subtype, age, gender)
Understood/ Able to describe	N=4	N=2	N=6	MCL, 60 years old, M: *They're fine. Yes, they're understandable.*
Did not understand	N=0	N=0	N=0	-
Unclear	N=0	N=0	N=0	-
Not asked	N=1	N=0	N=1	-
**Domain instructions**				
Understood/ Able to describe	N=3	N=2	N=5	DLBCL,53 years old, F: *They're clear.*
Did not understand	N=0	N=0	N=0	-
Unclear	N=0	N=0	N=0	-
Not asked	N=2	N=0	N=2	-
**Recall period**				
Understood/ Able to describe	N=5	N=1	N=6	DLBCL,55 years old, M: *I'm thinking since today's Wednesday, it would be going back to last Wednesday.*
Did not understand	N=0	N=0	N=0	-
Unclear	N=0	N=0	N=0	-
Not asked	N=0	N=1	N=1	-
**Response options**				
Understood/ Able to describe	N=5	N=2	N=7	DLBCL,58 years old, M: *Not at all means nothing. A little bit would be that I was aware of it. Somewhat means it's at a pivotal point. Then quite a bit, obviously that means that it's happening to me or very much would be, quite a bit is quite a, is the vast majority of the seven days. Very much would be, to me, mean it's affected me, again, all the time.*
Did not understand	N=0	N=0	N=0	-
Unclear	N=0	N=0	N=0	-

Parameters	DLBCL N=5	MCL N=2	Total N=7	Illustrative quotes (NHL subtype, age, gender)
Not asked	N=0	N=0	N=0	-
**EORTC QLQ-NHL-HG29**				
**Overall instructions**				
Understood/able to describe	N=5	N=2	N=7	DLBCL, 63 years old, F: *They are clear, I answered the same questions with the same garbage on the ones without any help, so yeah, it's okay.*
Did not understand	N=0	N=1	N=1	MCL, 49 years old, M: *Is that as a patient, I sometimes have symptoms or problems. I'm trying to figure out which ones are symptoms and problems, I don't know.*
Unclear	N=0	N=0	N=0	-
Not asked	N=0	N=0	N=0	-
Changes/Rewording recommended	N=2	N=1	N=3	MCL, 49 years old, M: *I would group the questions in the symptoms, and then another section for problems.*
**Recall period**				
Understood/ Able to describe	N=2	N=2	N=4	DLBCL, 60 years old, M: *So, it's basically asking, within the last past week or seven days, if I experience the following symptoms or problems.*
Did not understand	N=0	N=0	N=0	-
Unclear	N=0	N=0	N=0	-
Not asked	N=3	N=1	N=4	-
**Response options**				
Understood/ Able to describe	N=5	N=2	N=8	DLBCL, 63 years old, M: *[Not at all] Nothing happened, didn't happen.*
Did not understand	N=0	N=0	N=0	-
Unclear	N=0	N=0	N=0	-
Not asked	N=0	N=0	N=0	-

Parameters	DLBCL N=5	MCL N=2	Total N=7	Illustrative quotes (NHL subtype, age, gender)
**EORTC QLQ-NHL-LG20**				
**Overall instructions**				
Understood/ Able to describe	N=6	N=4	N=10	DLBCL, 62 years old, F: *Very clear.*
Did not understand	N=0	N=0	N=0	
Unclear	N=0	N=0	N=0	
Not asked	N=0	N=0	N=0	
**Domain instruction**				
Understood/ Able to describe	N=6	N=4	N=10	DLBCL,62 years old, F: *It's saying there's symptoms and problems, and, you know, mark what you've experienced during the past week. And circle the number that best applies to you.*
Did not understand	N=0	N=0	N=0	-
Unclear	N=0	N=0	N=0	-
Not asked	N=0	N=0	N=0	-
**Recall period**				
Understood/ Able to describe	N=5	N=4	N=9	DLBCL,62 years old, F: *The past week, past seven days.*
Did not understand	N=0	N=0	N=0	-
Unclear	N=0	N=0	N=0	-
Not asked	N=1	N=0	N=1	-
**Response options**				
Understood/ Able to describe	N=6	N=3	N=9	DLBCL, 56 years old, M: *Very much means it's bothering you—to me, that means all the time.*
Did not understand	N=0	N=0	N=0	-
Unclear	N=0	N=0	N=0	-
Not asked	N=0	N=1	N=1	-

DLBCL, diffuse large B-cell lymphoma; EORTC QLQ-C30, the European Organisation for Research and Treatment of Cancer Quality of Life Questionnaire–Core 30; EORTC QLQ-NHL-HG29, EORTC QLQ-High Grade Module 29; EORTC QLQ-NHL-LG20, EORTC QLQ-Low Grade Module 20; F, female; FACT-Lym, Functional Assessment of Cancer Therapy–Lymphoma; M, male; MCL, mantle cell lymphoma; PRO, patient-reported outcome.
